# Tropomodulin-3 is essential in asymmetric division during mouse oocyte maturation

**DOI:** 10.1038/srep29204

**Published:** 2016-07-04

**Authors:** Yu-Jin Jo, Woo-In Jang, Nam-Hyung Kim, Suk Namgoong

**Affiliations:** 1 Department of Animal Sciences, Chungbuk National University, Cheong-Ju, ChungChungBuk-do, Republic of Korea

## Abstract

The dynamic polymerization and depolymerization of actin filaments is essential for various cellular processes such as cell migration, rotation, cytokinesis, and mammalian oocyte maturation. Tropomodulin 3 (Tmod3) binds to the slow-growing (pointed) ends of the actin filament, thereby protecting the filament from depolymerization. However, the roles of Tmod3 in mammalian oocyte maturation remain elusive. Tmod3 mRNA and protein is present at all stages of mouse oocyte maturation. Tmod3 protein is mainly localized in the cytoplasm and appears enriched near the chromosome during maturation. By knocking down or ectopically overexpressing Tmod3, we confirmed that Tmod3 regulate the level of the intracytoplasmic actin mesh and asymmetric spindle migration. Expression of N-terminal Tmod3 (correspond to 1–155 amino acids), which contains the tropomyosin-binding site, results in decreased density of the actin mesh, thereby demonstrating the importance of the interaction between tropomyosin and tropomodulin for the maintenance of the actin mesh. Taken together, these findings indicate that Tmod3 plays crucial roles in oocyte maturation, presumably by protecting the actin filament from depolymerization and thereby controlling the density of the cytoplasmic actin mesh.

The dynamic reorganization of actin filaments is the main driving force for asymmetric spindle migration and polar body extrusion in mammalian oocytes^1^,^2^. The formation of the cortical actin cap near the spindle^3^ and changes in cytoplasmic actin mesh density^4^,^5^ represent two characteristic events occurring during the reorganization of the actin cytoskeleton in maturing oocytes. Various actin-binding proteins (ABPs) play essential roles in facilitating these changes in the actin cytoskeleton, as shown in the other biological systems^6^,^7^. For example, actin nucleators, such as formin-2 (FMN2)^8^,^9^, Spire^10^, and the Arp2/3 complex^11^,^12^,^13^, represent a family of proteins that initiate the formation of new actin filaments, thereby playing an important role in oocyte maturation.

In addition to actin nucleators, other ABPs such as the heterodimeric actin capping protein (CP)^14^ and tropomyosin^15^ have been recently reported to play roles in oocyte maturation^16^,^17^. For example, CP, which binds to the fast growing ends (also known as the barbed ends) of actin filaments to block further elongation, is considered a core protein in the regulation of actin cytoskeleton dynamics^18^. The roles of CP in oocyte maturation have been recently investigated^17^. Knockdown of CP expression in mouse oocytes results in significant impairment of spindle migration and asymmetric division of oocytes, as well as a decrease in cytoplasmic actin mesh level. In addition, the expression of the capping protein-binding region of the CARMIL protein which is known to exert decapping activity by reducing the affinity of CP for the barbed ends of actin filaments^19^,^20^,^21^, also decrease in the actin mesh level, indicating that CP is important for the maintenance of cytoplasmic actin mesh level during oocyte maturation. In addition to CP, tropomyosin (nonmuscle isoform Tpm3-1) is also shown to be important for oocyte maturation^16^. The impairment of Tpm3-1 expression by RNAi has been demonstrated to compromise asymmetric division in oocytes and severely impair oocyte cortical integrity, as evidenced by the formation of blebs during cytokinesis. It has also been shown that the decrease in cortical actin levels due to expression of dominant active cofilin can be rescued by coexpression of Tpm3-1, indicating that tropomyosin binds the sides of actin filaments, protects them from depolymerization by ADF/cofilin^15^,^22^ during oocyte maturation.

As demonstrated in these studies, the maintenance of cortical and cytoplasmic actin mesh is crucial for oocyte maturation. In addition to tropomyosin and CP, more than 100 actin binding proteins exist^23^; however, only a fraction of these proteins have been characterized in terms of their roles in oocyte maturation.

Tropomodulin is a ~40 kDa protein known to bind the slow-growing ends (pointed ends) of actin filaments and prevent depolymerization from the pointed ends^24^,^25^. Tropomodulin typically consists of an N-terminal tropomyosin-binding helix region, actin-binding region, and C-terminal leucine-rich repeat (LRR) regions^26^. The recent elucidation of the structure of the tropomodulin-actin complex reveals a detailed mechanistic view of pointed-end capping of tropomodulin^27^. Two separate actin-binding sites of tropomodulin, located at the N-terminal and C-terminal, bind two actin monomers separately on the pointed end of the actin filament, thereby blocking further elongation of the filament. In addition to interacting with the pointed end of the actin filament, tropomodulins interact with tropomyosin via two tropomyosin-binding sites located at their N-terminal regions^28^.

Four isoforms of tropomodulin, Tmod1, Tmod2, Tmod3, and Tmod4, exist in the genomes of mice and humans^25^. Several cellular and physiological functions of tropomodulins have been reported recently. For example, red blood cells from tropomodulin 1 (Tmod1)-null mice exhibit an abnormal sphero-elliptocytic shape, with reduced deformability and increased osmotic fragility^29^, demonstrating the role of Tmod1 in maintain membrane integrity. Depletion of tropomodulin (Tmod3) by siRNA in human microvascular epithelial cells enhances the rate of cell migration^30^, whereas the overexpression of Tmod3 is associated with impairment of cell migration. Tmod1-null skeletal muscle cells exhibit misaligned myofibrils and swelling of the sarcoplasmic reticulum^31^,^32^. In addition, Tmod1 plays an important role in the maintenance of hexagonal geometry in the fiber cells of the eye lens^33^,^34^. In the case of Tmod3 knockout mouse, deletion of Tmod3 caused the embryonic lethality at E14.5-E18.5, presumably by anemia caused by impairment of erythroid development^35^.

Cytoplasmic actin mesh formation is necessary for spindle migration and asymmetric cell division during maturation in mouse oocytes^5^,^36^,^37^. The formation of the actin mesh is mediated by formin2 and spire^5^,^10^. In contrast to Arp2/3 complex, which binds to the pointed ends of actin filaments, actin nucleators of the formin family bind to the fast-growing end of actin filaments^38^,^39^. Therefore, the pointed ends of actin filaments must be capped by pointed-end binding protein, in order to prevent depolymerization. Tropomodulin is the only known protein capable of capping the pointed ends of the actin filament. Previously, we have shown that knockdown of CP or partial abrogation of expression of CARMIL decreases cytoplasmic actin mesh levels in maturing oocytes^17^, indicating that actin capping is required for the maintenance of actin mesh filaments in oocytes. We investigated the functional roles of Tmod3 during oocyte maturation, and discovered that this protein is necessary for asymmetric division in oocytes via maintenance of the cytoplasmic actin mesh.

## Results

### Presence of Tmod3 protein in the oocyte cytoplasm during oocyte maturation

**Figure 1:**
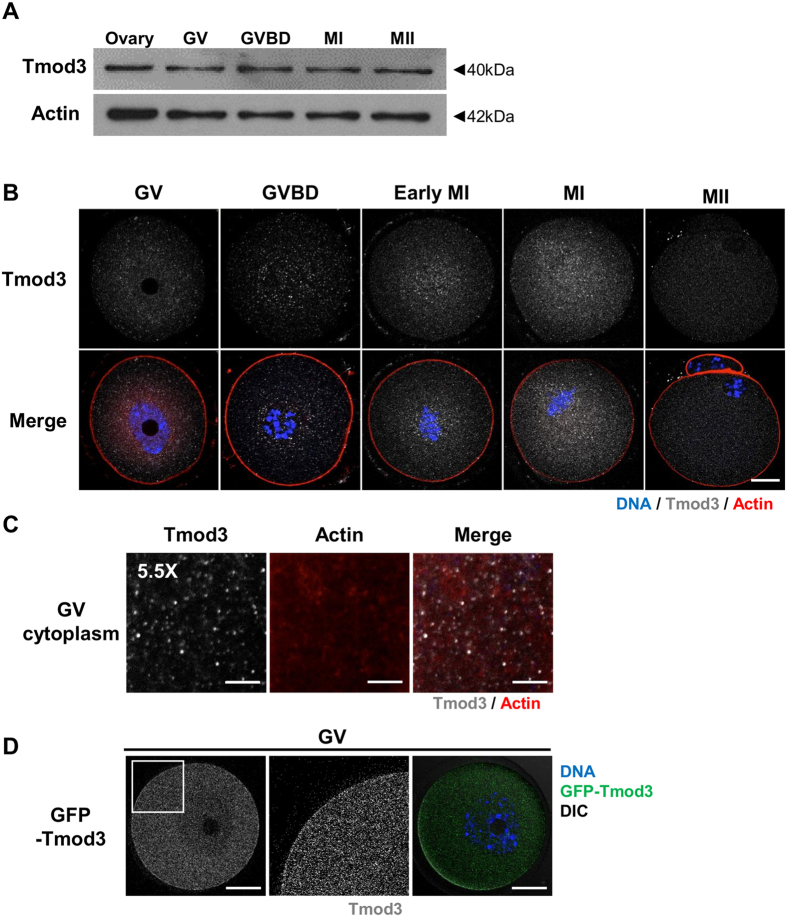
Expression and localization of Tmod3 during mouse oocyte maturation. (**A**) Western blot analysis of Tmod3 protein levels in ovarian tissue and GV, GVBD, MI and MII stage oocytes. A total of 100 oocytes at each stage were used. Tmod3, 40 kDa; Actin, 42 kDa. (**B**) Subcellular localization of Tmod3 during mouse oocyte meiotic maturation. Immunofluorescence staining was performed using an anti-Tmod3 antibody. Tmod3 was found to accumulate throughout the cytoplasm during oocyte maturation. Blue; DNA, red; actin, gray; Tmod3. Scale bar: 20 μm. (**C**) Punta-like localization of Tmod3 in the cytoplasm of GV-stage oocytes. Gray; Tmod3, red; actin. Scale bar: 4 μm. (**D**) Localization of GFP-Tmod3 in GV stage oocyte. 5 pl of 500 ng/μl GFP–Tmod3 mRNA was microinjected into oocyte. The white square in GV cytoplasm indicated was displayed as magnified view in the right.

Based on our previous finding that barbed-end actin-capping protein is essential for oocyte maturation^17^, we investigated the roles of the pointed-end capping protein Tmod3, an ubiquitous isoform of tropomodulin^40^, in mouse oocyte maturation. To date, there have been no reports on Tmod3 expression in mammalian gametes, including oocytes. Therefore, we examined the expression of Tmod3 at the mRNA and protein level using quantitative reverse-transcription PCR and western blotting. As shown in Supplementary Figure 1A, Tmod3 mRNAs are present at all stages of immature (germinal-vesicle stage; GV) oocytes, germinal vesicle breakdown (GVBD), and metaphase I (MI) and metaphase II (MII) oocytes (Supplementary Figure 1A). Then, we investigated the presence of Tmod3 protein in oocytes by western blotting (Fig. 1A). Tmod3 proteins, around 40 kDa in size, were detected at all stages of oocyte maturation. Moreover, their levels remained constant throughout maturation. Next, we investigated the localization of Tmod3 in the oocyte at various stages of maturation using immunostaining with Tmod3 antibody. At all developmental stages, Tmod3 was dispersed throughout the entire cytoplasm of the oocyte in speckle form (Fig. 1B,C). To confirm Tmod3 localizations, we expressed GFP-Tmod3 in oocytes. As similar with immunostaining results, GFP-Tmod3 is dispersed in cytoplasm (Fig. 1D and Supplementary Figure 1B,C). While Tmod3 localizations are not observed in immunostaining results, faint localizations of GFP-Tmod3 is also observed at the cortex. Taken together, these results confirm the expression of Tmod3 in maturing mouse oocytes and localized mainly in the cytoplasm.

### Knockdown of Tmod3 impairs the asymmetric division of oocytes and decreases cytoplasmic actin mesh and cortical actin levels

Previously, we showed that knockdown of barbed ends binding CP or overexpression of CP-binding region (CBR) in CARMIL lowers cytoplasmic actin mesh levels, thereby impairing spindle migration and asymmetric division in mouse oocytes^17^. We tested whether the ablation of Tmod3, which is known to cap the pointed ends of actin filaments, affects polar body extrusion or cytoplasmic actin mesh density during oocyte maturation, as observed for CP.

**Figure 2:**
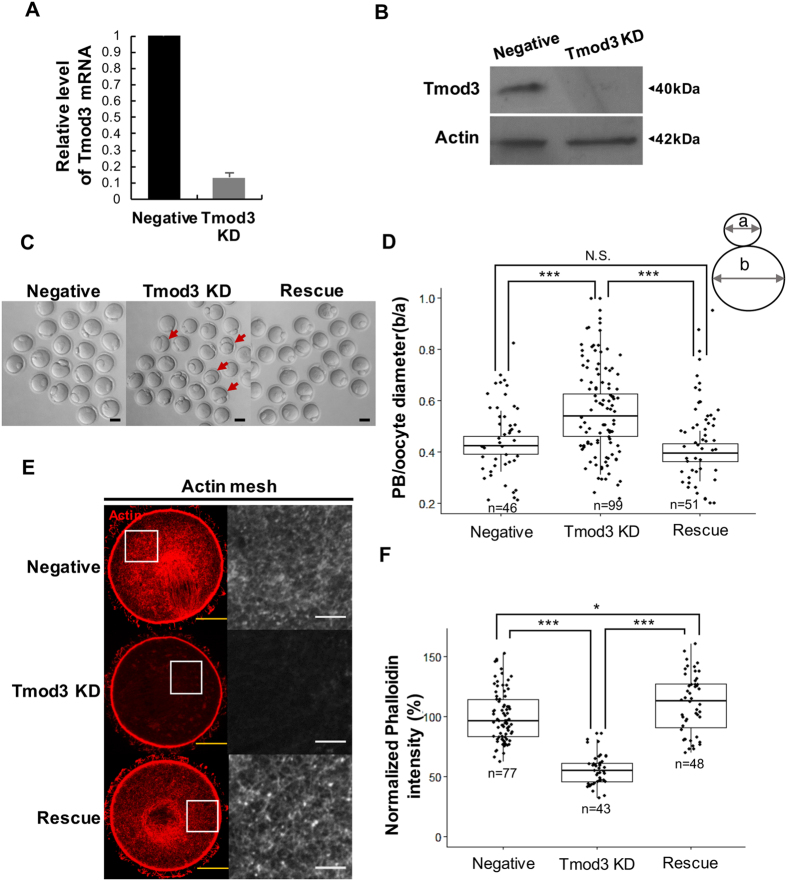
Tmod3 is essential for asymmetric division during oocyte maturation. (**A**) Knockdown of Tmod3 mRNA by dsRNA injection. mRNA levels in dsRNA-injected oocytes (n = 30) are expressed relative to those in negative control. Data indicate the mean ± S.E.M. for three independent experiments. (**B**) Tmod3 protein depletion by dsRNA injection. Western blotting of Tmod3 showing depletion of Tmod3 protein levels by dsRNA against Tmod3 (Tmod3 KD) compared with control (Negative) at the GV stage. (**C**) Impairment of asymmetric division by knockdown of Tmod3. Arrows indicate abnormal polar body extrusions. Scale bars: 50 μm (**D**) Tmod3 knockdown increases polar body (PB) size. The distribution of PB diameters expressed as ratio of diameter to that of the oocyte in control, rescue, and Tmod3- knocked down oocytes is shown. n values are as indicated. Boxes show the interquartile range; whiskers show 1.5× the interquartile range; line represents the median. ***P < 0.001; N.S.: not statistically significant (P > 0.05). (**E**) Phalloidin-stained cytoplasmic actin mesh levels in oocytes injected with negative control dsRNA (Negative), Tmod3-specific dsRNA (Tmod3 KD), and cRNA encoding human Tmod3 (Rescue) at the MI stage. The portion of cytoplasm in the oocyte indicated in the square was displayed as magnified view in the right. Scale bars: 20 μm (Yellow), 3 μm (White). (**F**) Quantification of phalloidin fluorescence intensity of cytoplasmic actin at 8 h (MI) after meiosis resumption in oocytes injected by negative control dsRNA (Negative), Tmod3-specific dsRNA (Tmod3 KD), and cRNA encoding human Tmod3 (Rescue). Phalloidin intensities of oocytes were normalized to the mean intensity of control oocytes and presented as percent (%) of intensity relative to mean of control. Oocyte number used for the analysis was indicated as n. Boxes show the interquartile range; whiskers show 1.5× the interquartile range; line represents the median. ***P < 0.001; *P < 0.05.

Knockdown of RNA expression levels to 13% of that of negative control-injected oocytes was achieved by injecting double-strand RNA (dsRNA) against the Tmod3 gene (Fig. 2A). We confirmed that the injection of Tmod3 dsRNA results in a depletion of Tmod3 protein using by western blot and immunostaining, as shown in Fig. 2B and Supplementary Figure 1D,E. Next, we examined the effect of Tmod3 knockdown on oocyte maturation. The percentage of oocytes that reached MII stage did not significantly differ between the control and Tmod3 knockdown groups (Supplementary Figure 1F). However, oocytes with knockdown of Tmod3 frequently exhibited an abnormally large polar body, or underwent symmetric division. It was found that the co-injection of RNAi-resistant human Tmod3 rescued these defects (Fig. 2C). On examining the ratio of the size of the polar body to that of the oocyte, we found a significant increase in the ratio of polar body diameter to oocyte diameter. However, in oocytes in which Tmod3 knockdown had been rescued, no significant difference was observed relative to the control group (Fig. 2D), confirming that the failure of spindle migration was caused by Tmod3 knockdown.

In order to elucidate the mechanisms underlying the failure of asymmetric division in Tmod3 knockdown oocytes, we investigated the effect of Tmod3 knockdown on cytoplasmic actin mesh levels, which are crucial for spindle migration and oocyte maturation^5^,^10^,^36^. Following microinjection of Tmod3 dsRNA, oocytes at MI stage were stained with phalloidin and the cytoplasmic actin mesh level was measured (Fig. 2E). Tmod3 dsRNA injections resulted in significantly lower cytosplasmic actin mesh level than that observed in the control dsRNA-injected group, whereas co-injection with dsRNA-resistant human Tmod3 cRNA rescued the actin mesh level (Fig. 2F). We also measured the effect of Tmod3 knockdown on cortical actin stability using fluorescence recovery after photobleaching (FRAP), using mCherry-UtrCH as probe for actin. As shown in the Supplementary Figure 2A–C there is no significant difference in fluorescence recovery between Tmod3 knockdown and negative control control injected groups (See also Supplementary movie 1 and 2). Collectively, these data indicate that the knockdown of Tmod3 impairs asymmetric division and decreases cytoplasmic actin mesh and cortical actin thickness. These phenotypes are very similar to those observed in oocytes knocked down for CP expression^17^.

### Knockdown of Tmod3 impairs the gradual increase in the level of cytoplasmic actin during oocyte maturation and negatively affects spindle migration

**Figure 3:**
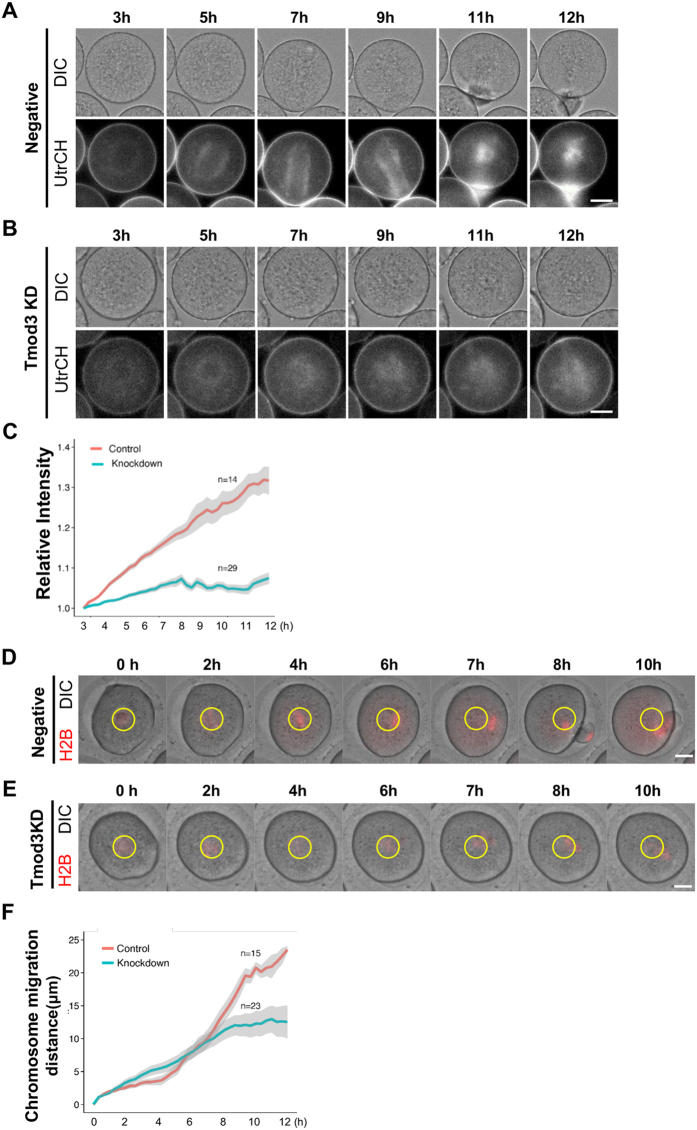
Knockdown of Tmod3 impairs the change in cytoplasmic actin levels and spindle migration during oocyte maturation. (**A–C**) Changes in cytoplasmic actin levels in maturing negative control (**A**) and Tmod3-knockdown (**B**) oocytes. Filamentous actin was visualized by injection of cRNA encoding GFP-fused UtrCH. See also Supplementary movies 3 and 4. Scale bars: 20 μm. (**C**) Time course of cytoplasmic actin levels during oocyte maturation. Red; control oocytes injected with GFP-UtrCH, Cyan; oocytes injected with Tmod3 dsRNA as well as GFP-UtrCH. Fluorescence Intensities of cytoplasm devoid of cortical regions in oocytes were expressed as relative values based on the those in the first frame. Normalized fluorescence intensities of control (n = 14) or Tmod3 dsRNA (n = 29) oocytes at each time point were averaged and S.E.M of each time point was indicated as envelope. (**D–F**). Chromatin tracking of maturing control (**D**) and Tmod3-knockdown (**E**) oocytes. Chromatin was visualized by injection of cRNA encoding H2B–mCherry (red). The initial locations of chromatin at GV stages are marked with yellow ovals. See also Supplementary movies 5 and 6. Scale bars: 20 μm. (**F**) Time course of chromosome tracking in negative control (Red) and Tmod3-knockdown (cyan) oocytes. The distances (μm) of the chromosome from their starting points at each time point in control (n = 15) and Tmod3 knockdown (n = 23) oocytes were averaged and S.E.M for each time point was indicated as envelope.

In order to further characterize phenotypes mediated by Tmod3, we measured dynamic changes in cytoplasmic actin filaments by microinjecting oocytes with the actin probe GFP-UtrCH1^41^. In control oocytes injected with only GFP-UtrCH, fluorescence levels steadily increased during maturation (n = 14), as shown in Fig. 3A,C and Supplementary Movie 3. This finding is consistent with previous reports^5^. Tmod3 knockdown was associated with a much slower increase in fluorescence intensity (n = 29), which is in agreement with actin mesh quantification by phalloidin staining (Fig. 3B,C and Supplementary Movie 4). In addition, we tracked spindle movement by time-lapse imaging of chromatin, as visualized by H2B-mCherry fluorescence. As shown in Fig. 3D and Supplementary Movie 5, the spindle moved from the center of the oocyte to near the cortex in control oocytes; however, the movement of the spindle in Tmod3-knocked down oocytes is far smaller than those of control (Fig. 3E and Supplementary Movie 6). Based on the average movement of chromatin in control oocytes (n = 15) and oocytes knocked down for Tmod3 (n = 23), it is evident that spindle migration speeds increase after 6 h in control oocytes, whereas this rapid migration of spindles is not observed in the Tmod3-knockdown group (Fig. 3F).

Actin-rich membrane blebs are usually associated with actin cytoskeleton collapse in cell cortex^42^. Previously we reported that knockdown of Tpm3.1 in mouse oocytes caused blebs formations during maturation^16^. In the case of Tmod3-knockdown group, several oocytes formed membrane blebs during maturation (8/38 of oocyte), while control oocytes did not (0/37) (Supplementary Figure 3). Collectively, these results show that the knockdown of Tmod3 causes impairment of cytoplasmic actin mesh and impairs spindle migration.

### Tropomodulin 3 and Tropomyosin 3 both contribute for the maintenance of cytoplasmic actin mesh

It is known that Tmod3 has two binding sites for tropomyosin, and that the interaction between tropomodulin and tropomyosin enhances pointed-end binding affinity^27^,^43^. Moreover, antibodies specific for the tropomyosin-binding regions of Tmod1 result in unstable pointed ends of actin filaments by dissociating tropomyosin from these ends^44^, suggesting that the interplay between tropomodulins and tropomyosin is crucial for their roles as actin- binding proteins.

**Figure 4:**
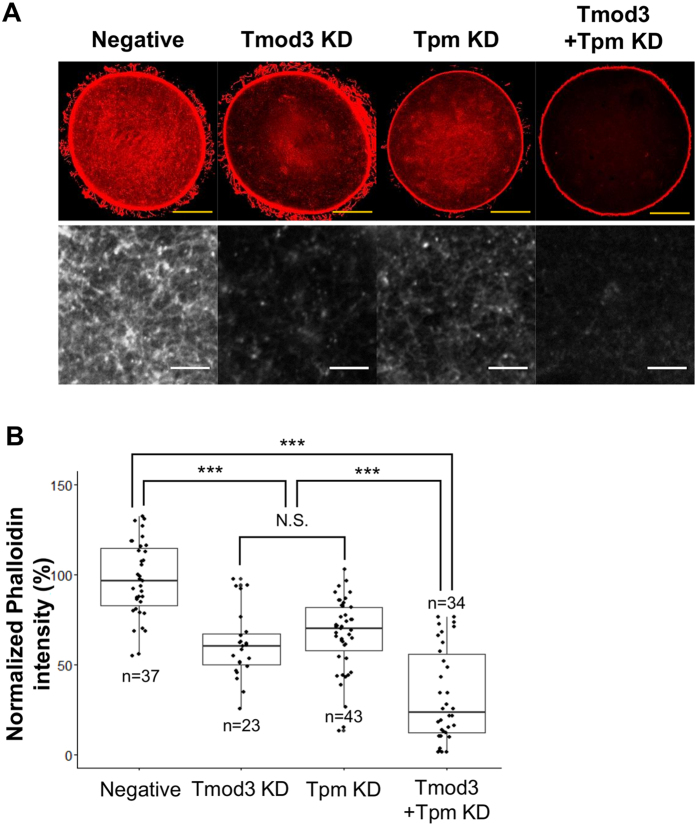
Interplay of Tmod3 and Tpm3 in the regulation of cytoplasmic actin mesh. (**A**) Phalloidin-staining of the cytoplasmic actin mesh in oocytes injected with negative control dsRNA (Negative), Tmod3-specific dsRNA (Tmod3 KD), Tpm3-specific siRNA (Tpm KD), and Tmod3 dsRNA + Tpm3 siRNA (Tmod3 + Tpm KD) at the MI stages under the same conditions. Scale bars: 20 μm (Yellow), 3 μm (White). (**B**) Quantification of phalloidin fluorescence intensity of cytoplasmic actin at 8 h (MI) after meiosis resumption in oocytes injected by dsRNAs. Phalloidin intensities of each oocytes were normalized to the mean intensity of control oocytes and presented as percent (%) of intensity relative to mean of control. Three independent experiments were carried out. Oocyte number used for the analysis was indicated as n. Boxes show the interquartile range; whiskers show 1.5× the interquartile range; line represents the median. ***P < 0.001; *P < 0.05.

Previously, we reported that knockdown of Tpm3.1 causes a decrease in cortical actin levels^16^; however, the effect on cytoplasmic actin mesh levels has not been investigated. As the knockdown of Tmod3 decreases actin mesh levels significantly (Fig. 2), we reasoned that Tpm3.1 knockdown might affect the levels of cytoplasmic actin mesh as well as those of cortical actin. We checked the actin mesh levels in oocytes in which Tmod3 and Tpm3.1 had been separately knocked down and compared these with actin mesh levels in the control. First we confirmed Tpm3.1 knockdown by siRNA injections (Supplementary Figure 4). We found that knockdown of Tpm3.1 decreases actin mesh levels significantly, similarly to Tmod3 knockdown (Fig. 4A,B). Knockdown of both Tmod3 and Tpm3.1 was found to elicit a greater decrease in the actin mesh level than separate knockdown of Tmod3 or Tpm3.1 (Fig. 4A,B), indicating that the negative effect of Tmod3 and Tpm3.1 on actin mesh levels is additive and suggesting a synergy between Tmod3 and Tpm3.1 in the maintenance of actin mesh levels.

### Overexpression of full length or N-terminal half of Tmod3 impair asymmetric division by affecting cytoplasmic actin mesh levels

**Figure 5:**
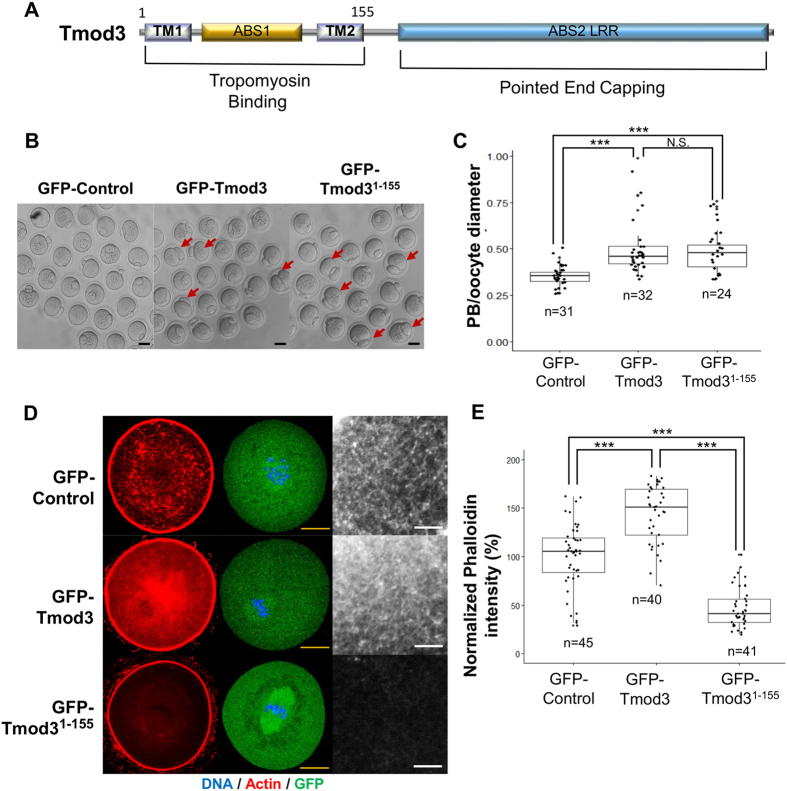
Overexpression of Tmod3 promotes the increase of cytoplasmic actin mesh levels, whereas expression of N-terminal region exerts dominant-negative effects. (**A**) Domain structure of Tmod3. Tmod3 consists of two tropomyosin-binding sites (TM1 and TM2, respectively) and two actin-binding sequences (ABS1 and ABS2). The second ABS2 is a leucine-rich repeat (LRR). The full-length Tmod3 construct (GFP-Tmod3) contains all the functional domains of the Tmod3 protein; however, Tmod3^1-155^ only contains two tropomyosin-binding sites and one actin-binding site. (**B**) Impairment of asymmetric division by overexpression of Tmod3 and GFP-Trmod3^1-155^. After GFP-control, GFP-Tmod3, and GFP-Trmod3^1-155^ mRNA injection and maturation arrest for 3 h to ensure that mRNA was expressed, maturation was resumed and samples were collected 12 h later. Arrows indicate abnormal polar body extrusions. Scale bars: 50 μm (**C**) Tmod3 full length and GFP-Trmod3^1-155^ overexpression increases polar body (PB) size. The distribution of PB diameters expressed as ratio of diameter to that of the oocyte in GFP-control, GFP-Tmod3, and GFP-Trmod3^1-155^ overexpressed oocytes is shown. n values are as indicated. Boxes show the interquartile range; whiskers show 1.5× the interquartile range; line represents the median. ***P < 0.001; N.S.: not statistically significant (P > 0.05). (**D**) Phalloidin-staining of the cytoplasmic actin mesh in oocytes injected with GFP mRNA (GFP-Control) or GFP–Tmod3 full length mRNA (GFP-Tmod3) or GFP-Trmod3^1-155^ and then matured to MI (8 h) stages. Red; phalloidin-stained actin, green; GFP, blue; DNA. Scale bars: 20 μm (Yellow). Right panel shows actin channel with different magnifications. Scale bars: 3 μm (White). (**E**) Quantification of phalloidin fluorescence intensity of cytoplasmic actin at 8 h (MI) after meiosis resumption in oocytes injected by cRNAs. Phalloidin intensities of each oocytes were normalized to the mean intensity of control oocytes and presented as percent (%) of intensity relative to mean of control. Three independent experiments were carried out. Oocyte number used for the analysis was indicated as n. Boxes show the interquartile range; whiskers show 1.5× the interquartile range; line represents the median. ***P < 0.001; *P < 0.05.

The domain structures of Tmod3 reveal that this protein consists of an N-terminal region, which binds two tropomyosin molecules, and C-terminal Leucine-Rich-Repeat (LRR) regions, which bind the pointed ends of actin filaments (Fig. 5A).

In order to confirm that Tmod3 protects the pointed ends of actin filaments in the cytoplasmic actin mesh, and to demonstrate direct interactions between tropomyosin and Tmod3, we designed two Tmod3 constructs. The first one corresponded to the full-length Tmod3 protein fused with N-terminal GFP (GFP-Tmod3). The other corresponded to the N-terminal half (amino acids 1–155) of Tmod3, which contained two tropomyosin-binding site (TM1 and TM2) and one actin-binding site (ABS1).

The percentage of oocytes that occurred cytokinesis did not significantly differ between the GFP-control and GFP-Tmod3 overexpression groups (Supplementary Figure 1G). However, Overexpression of the full-length Tmod3 or Tmod3_1-155_ resulted in an abnormally large polar body, or underwent symmetric division, as similar with phenotype observed in Tmod3 knockdown groups (Fig. 5B,C), indicating that overexpression of full-length Tmod3 or Tmod3_1-155_ also impair spindle migrations. We checked the effects of Tmod3 constructs overexpression on cytoplasm actin mesh density. While full-length Tmod3 overexpressed oocytes have significantly higher cytoplasmic actin mesh levels than in the controls (Fig. 5D,E), indicating that overexpression of Tmod3 increases the stability of cytoplasmic actin mesh. In contrast, overexpression of Tmod3_1-155_, which contains tropomyosin-binding sites, but lacks the pointed end-capping LRR domain, resulted in a decreased cytoplasmic actin mesh level compared with control oocytes, indicating that the expression of N-terminal regions of Tmod3 elicits dominant-negative effects on actin mesh levels.

### Overexpression of Formin2 fails to maintain a high level of cytoplasmic actin mesh density in the absence Tropomodulin-3

**Figure 6:**
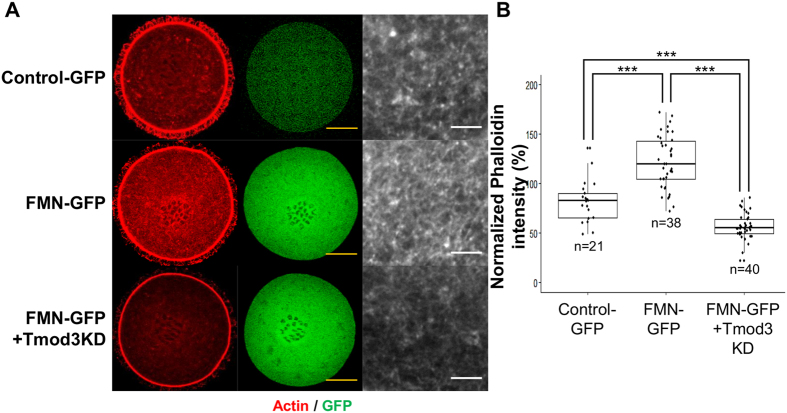
Overexpression of Formin-2 (FMN2) fails to maintain high levels of cytoplasmic actin mesh density in the absence of Tmod3. (**A**) Phalloidin-staining of the cytoplasmic actin mesh in oocytes injected with mRNA encoding GFP (Control-GFP) or GFP-FMN2^FH1FH2^ (FMN-GFP) or GFP-FMN2^FH1FH2^ and dsRNA against Tmod3 (FMN-GFP + Tmod3 KD) at MI stage. Scale bars: 20 μm (Yellow). The figure on the right indicates magnification of the cytoplasm of oocytes in each group. Scale bars: 3 μm (White). (**B**) Quantification of phalloidin fluorescence intensity of cytoplasmic actin at 8 h (MI) after meiosis resumption in oocytes injected by cRNAs. Phalloidin intensities of each oocytes were normalized to the mean intensity of control oocytes and presented as percent (%) of intensity relative to mean of control. Three independent experiments were carried out. Oocyte number used for the analysis was indicated as n. Boxes show the interquartile range; whiskers show 1.5× the interquartile range; line represents the median. ***P < 0.001; *P < 0.05.

It is known that the actin mesh in the oocyte cytoplasm is generated by formin-2^5^ and spire^10^ considering that the actin nucleators of the formin family elongate actin filaments and bind to their fast-growing ends^38^, capping of the pointed ends of filaments by tropomodulin may be necessary to prevent depolymerization. In order to demonstrate the importance of pointed-end capping in the maintenance of cytoplasmic actin mesh levels, we overexpressed Formin2 (FH1-FH2 domains of FMN2 gene) as a GFP fusion protein and examined its effects on cytoplasmic actin mesh. Consistent with previous results^17^,^36^, the expression of GFP-FMN2 (FH1FH2) results in higher actin mesh levels compared with those in the GFP control group. However, when dsRNA against Tmod3 is co-injected with GFP-FMN2 (FH1FH2) cRNAs, the actin mesh level is significantly lower than that in GFP-FMN2 (FH1FH2) as well as in the GFP control (Fig. 6A,B). These results indicate that FMN2, even at high levels, is unable suppress the effect of Tmod3 in decreasing actin mesh levels, highlighting the importance of Tmod3 in the maintenance of actin mesh levels.

## Discussion

In this study, we demonstrated that Tmod3 is required for the maintenance of the cytoplasmic actin mesh and during oocyte maturation and failure of maintenance of proper actin mesh levels impair spindle migration. We additionally showed that the interplay between Tmod3 and Tpm3 is crucial for their activity. Since the importance of dynamic actin reorganization in oocyte maturation became known to the community, several studies have focused on the roles of actin nucleators such as formin-2^8^,^9^, spire^10^, the Arp2/3 complex^11^, and NPFs, which activate the Arp2/3 complex—for example, WAVE2^45^, JMY^46^, N-WASP^47^ and WASH^48^. However, far less attention has been paid to the roles of other actin-binding proteins in oocyte maturation. As in previous studies on the roles of CP^17^ and tropomyosin^16^, we showed that the control of post-nucleated actin filaments by actin cytoskeleton remodeling is also important for meiotic maturation, as already shown in other cellular phenomena such as cell migration^6^ and cytokinesis^49^.

In this study, we demonstrated the ablation of spindle migration and failure of asymmetric division following the knockdown of Tmod3. These phenotypes may be linked with a decrease in cytoplasmic actin mesh levels observed during oocyte maturation, as shown by the measurement of the levels of phalloidin-stained actin mesh and time-lapse imaging of maturating oocytes/Formin-2/spire are known to be responsible for the generation of actin filaments in the cytoplasmic actin mesh in oocytes^8^,^9^,^10^. Moreover, formin-2 is a member of the formin-family proteins which nucleate and elongate actin filaments by binding to their fast-growing barbed ends^38^,^39^,^50^,^51^. The opposite site of actin filaments, slow-growing pointed ends need to be protected from depolymerization as the critical concentration of actin polymerization at the pointed end of the filaments is over 10-fold higher than that at the barbed ends^52^. Our results confirm the importance of pointed-end capping proteins in the maintenance of actin mesh during oocyte maturation. As shown in Fig. 6, even increased formin-2 levels are not sufficient to maintain cytoplasmic actin mesh in oocytes knocked down for Tmod3, indicating that both pointed-end capping and protection by Tmod3, as well as presence of the actin nucleator, are essential for the maintenance of the cytoplasmic actin mesh.

It is well established that tropomodulin binds tropomyosin, and that the presence of tropomyosin significantly enhances pointed-end capping activity of Tropomodulin^27^,^43^,^53^. Therefore, we investigated the functional roles of the interaction between Tmod3 and Tpm3.1, which is also reported to be important for oocyte maturation via maintenance of cortical integrity. The results of the present study indicate that pointed-end capping of Tmod3 and filament protection by Tpm3.1 are tightly coupled due to interaction between the two proteins. In addition to the additive negative effects of Tmod3 and Tpm3.1 knockdown on cytoplasmic actin mesh levels, the expression of the N-terminal region of Tmod3, which contains two tropomyosin-binding sites, exerts dominant-negative effects on the actin mesh level, presumably by sequestering of Tpm3.1 via tropomyosin binding region in N-terminal half of Tmod3. These results show that tropomyosin-tropomodulin interaction is essential for the maintenance of the actin mesh during oocyte maturation.

**Figure 7:**
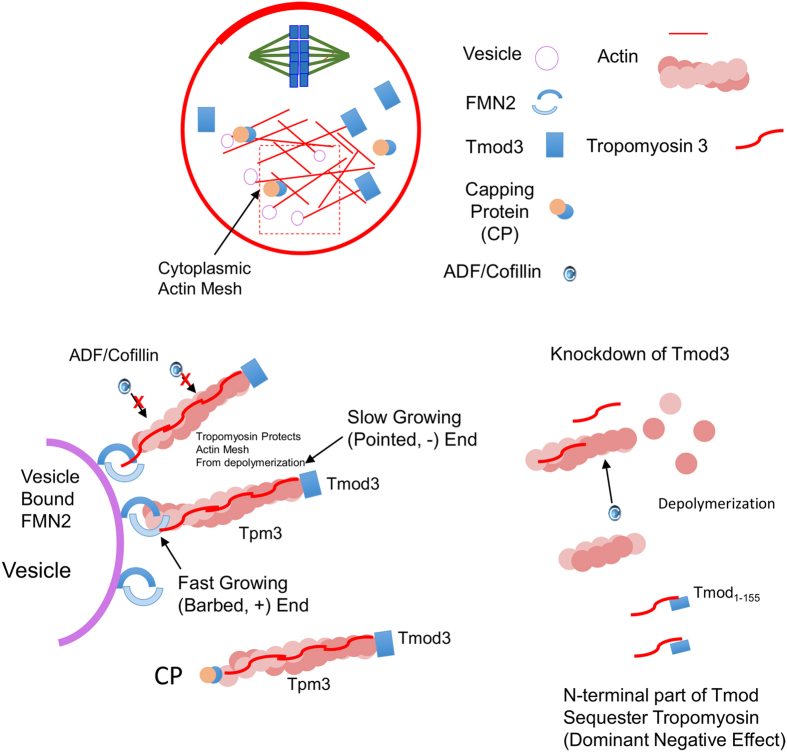
Suggested model for the roles of Tmod3, Tpm3, and CP in the cytoplasmic actin mesh. Cytoplasmic actin mesh is nucleated by formin-2 (FMN2) and spire localized on vesicles and cortex in oocyte^4^,^5^,^10^,^36^,^69^. Formin-2 moves along with the fast-growing barbed end. The pointed end of the actin filament, initiated by formin-2, is capped by Tmod3 and protected from depolymerization. Interaction between Tpm3 and Tmod3 enhances the pointed-end capping activity of Tmod3 and binding affinity of Tpm3, thereby protecting actin filaments from depolymerization, presumably via the actin-severing protein cofilin. Knockdown of Tmod3 results in depolymerization of the actin filament, thereby decreasing the concentration of the cytoplasmic actin mesh. Overexpression of the N-terminal region of Tmod3 containing tropomyosin-binding sites, results in sequestration of Tpm3, thereby causing dominant-negative effects.

Based on the results of the present study, we suggested a hypothetical model for the roles of Tpm3 and Tmod3 in the maintenance of the cytoplasmic actin mesh in maturing oocytes (Fig. 7). The pointed ends of actin filaments are capped by Tmod3, whose binding is facilitated by the binding of Tpm3 to the actin filament. Tpm3 binding also protects actin filaments from depolymerization by ADF/cofilin. CP binds to the barbed ends of actin filaments, thereby preventing further elongation of filaments or depolymerization^17^. A decrease in, or abrogation of, Tmod3 levels results in depolymerization of pointed ends, thereby decreasing actin mesh levels.

Considering the roles of Tmod3 and Tpm3 in the maintenance of the cytoplasmic actin mesh, the modulation of the actin-capping activity of Tmod3 may represent a potential regulatory mechanism controlling the amounts of actin mesh in the cell. However, it is not clear as to how Tmod3 activity is regulated. A recent report shows that Tmod3 is an effector of Akt2, and that phosphorylation of Tmod3 at Ser71 in its first actin-binding region regulates GLUT4 exocytosis via actin remodeling^54^. This suggests the possibility of a regulatory pathway of Tmod3 involving Akt in mammalian oocytes. Akt has been reported to be involved in mammalian oocyte maturation^53^,^55^,^56^,^57^,^58^. Additionally, the inhibition of Akt activity impairs meiotic maturation or spindle formation^59^. However, its effect on the actin cytoskeleton and possible links with tmod3 Tmod3 remained to be investigated.

Previously, various functional roles of tropomodulins have been reported. For example, it has been shown that the knockdown of Tmod3 in epithelial cells causes altered cell morphology, presumably due to decreased F-actin content in lateral membranes^60^. Additionally, it has been shown mouse lens fiber cells in Tmod1- knockout mice show disruption of spectrin-actin links and abnormal morphology^34^. The present study is the first demonstration describing the roles of Tropomodulin in gamete formation. Collectively, our data demonstrate the importance of pointed-end capping protein for maintenance of proper asymmetric division in mammalian oocytes. Further studies are required to elucidate the potential roles of these actin-binding proteins in early embryogenesis.

## Materials and Methods

### Antibodies

Goat polyclonal antibody against human Tmod3 (G-16, sc-19205) was acquired from Santa Cruz Biotech (Santa Cruz, CA, USA). A mouse monoclonal antibody against Tpm3 (CG3)^61^ was obtained from the Developmental Study Hybridoma Bank at the University of Iowa. An Alexa-Fluor-488-conjugated rabbit anti-goat-IgG antibody was purchased from Invitrogen (Carlsbad, CA, USA), and a mouse monoclonal anti-α-tubulin–FITC antibody was obtained from Sigma (St Louis, MO, USA).

### Oocyte collection and culture

All animal manipulations were approved and conducted according to the guidelines of the Animal Research Committee of Chungbuk National University (approval no. CBNUR-889-15-02). Germinal vesicle (GV)-intact oocytes were collected from the ovaries of 6–8-week-old imprinting control region (ICR) mice and cultured in M16 medium (Sigma) under paraffin oil at 37 °C in 5% CO_2_. Oocytes were collected for immunostaining and microinjection after culturing for various amounts of time.

### Real-time quantitative PCR analysis

Tmod3 expression was analyzed by real-time quantitative PCR (qRT-PCR) and the ΔΔCT method^62^. Total RNA was extracted from 30 oocytes using a Dynabead mRNA DIRECT Kit (Life Technologies, Foster City, CA). First-strand cDNA was generated using a cDNA Synthesis Kit (Takara, Kyoto, Japan) and Oligo (dT)_12–18_ primers. The PCR primers used to amplify Tmod3 are listed in Table 1. qRT-PCR was performed with SYBR Green in a final reaction volume of 20 μl (qPCR kit, Finnzymes, Vantaa, Finland). PCR conditions were as follows: 94 °C for 10 min, followed by 39 cycles of 95 °C for 15 s, 60 °C for 15 s, and 72 °C for 45 s, with a final extension step at 72 °C for 5 min. Finally, relative gene expression was quantified by normalization to the level of GAPDH mRNA. Experiments were conducted in triplicate.

### Preparation of dsRNA and cRNA

Tmod3 dsRNA was generated as described previously^17^. Briefly, a 604 bp fragment of the TMOD3 gene (corresponding to 644–1248 bp of NM_016963.2) was amplified from first-strand cDNA, which was generated from RNA extracted from MII oocytes using gene-specific primers containing the T7 promoter sequence (Supplementary Table S1). *In vitro* transcription was performed using a Megashortscript T7 Kit (Life Technologies). For overexpression of the TMOD3 gene, synthetic DNA fragments corresponding to the ORF of the mouse TMOD3 sequence were synthesized by Bioneer Co (Daejun, Korea) and subcloned as a GFP fusion into the pCS2+ vector^63^. For the expression of the N-terminal region (corresponding to amino acids 1–155 of Tmod3), the corresponding sequence of the TMOD3 gene was amplified by PCR and subcloned as an N-terminal GFP fusion.

For the expression of the FH1-FH2 domains of FMN2, cDNA spanning 811–1579aa of FMN2 proteins were amplified from mouse FMN2 cDNA clone (Obtained from GE Dharmacon), then subcloned into pRN3 vector^64^ as C-terminal GFP fusion. The pRN3-H2B-mCherry plasmids^65^ were kindly provided by Dr. JungSoo Oh (Sung Kyun Kwan University, Suwon, Korea). For *in vitro* transcription, a mMessage mMachine SP6 or T3 Kit (Life Technologies) was employed. In some cases, poly-A tails were added using a poly-A Tailing Kit (Life Technologies).

### Microinjection

siRNA, dsRNAs and cRNAs were microinjected into GV-stage mouse oocytes, as described previously^17^. TMOD3 knockdown was achieved by diluting TMOD3 dsRNA to obtain a final concentration of 1 mg/ml and microinjecting it into the cytoplasm of a fully grown GV-stage oocyte using an Eppendorf Femto Jet (Eppendorf AG, Hamburg, Germany) and a Nikon Diaphot ECLIPSE TE300 inverted microscope (Nikon UK Ltd, Kingston upon Thames, Surrey, UK) equipped with a Narishige MM0-202N hydraulic three-dimensional micromanipulator (Narishige Inc., Sea Cliff, NY). After injection, oocytes were incubated in M16 medium containing 5 mM milrinone for 20 hr to ensure knockdown. The oocytes were then transferred into fresh M16 medium and cultured for a further 12 h. The developmental stages of the oocytes were visualized by DAPI staining. Control oocytes were microinjected with 5–10 pl of dsRNA against GFP. In the case of TPM3.1 knockdown, Approximately 5–10 pl of 50 μM TPM3.1-targeting siRNA (5′-CAGCUUUGGU GUCUUUGAA (dTdT)-3′) corresponding to 1499–1517 bp of NM_001253738.1, Bioneer) was used as described previously^16^.

Polar body extrusion and cytokinesis were observed using a stereomicroscope. For rescue experiments, cRNAs encoding dsRNA-resistant human Tmod3 were added to the dsRNA at a concentration of 0.1 mg/ml. In order to microinject FMN2-GFP cRNAs, the concentrations of cRNA were adjusted to 1 mg/ml. Approximately 5–10 pl of cRNA was microinjected into the cytoplasm of a fully-grown GV-stage oocyte. After injection, oocytes were cultured in M16 medium containing 5 mM milrinone for 3 hr, then oocytes were washed five times in milrinone-free M16 medium for 2 min each time. The oocytes were then transferred into fresh M16 medium and cultured under paraffin oil at 37 °C in an atmosphere of 5% CO_2_ in air. Control oocytes were microinjected with 5–10 pl of GFP cRNA. For the quantification of oocyte and polar body diameter, image of oocytes after *in vitro* maturation were acquired using Nikon Diaphot ECLIPSE TE300 inverted microscope. The longest diameters of oocytes and polar bodies were measured using ImageJ.

### Immunostaining and confocal microscopy

For immunostaining of Tmod3 or Tpm3-1, oocytes were fixed in 4% paraformaldehyde dissolved in phosphate-buffered saline (PBS) for 30 min at 20 °C, and then transferred into a membrane permeabilization solution (0.5% Triton X-100 in 0.1% polyvinylalcohol in phosphate-buffered saline, PVA-PBS) and incubated for 1 h. For the quantitative measurement of cortical and cytoplasmic actin mesh, membrane permeabilization step was skipped to preserve actin density. After 1 h in blocking buffer (PBS containing 1% BSA), the oocytes were incubated overnight at 4 °C with a goat anti-Tmod3 antibody at a 1:200 dilution. After three washes in washing buffer (PBS containing 0.1% Tween 20 and 0.01% Triton X- 100), the oocytes were labeled with Alexa-Fluor-488-conjugated goat anti-mouse-IgG (1:100) for 1–2 h at room temperature. In order to stain cytoplasmic actin mesh or cortical actin, oocytes were fixed and stained with phalloidin–TRITC (10 mg/ml), which labels F-actin. For anti-α-tubulin–FITC (1:200) antibody staining, oocytes were incubated for 1 h, washed three times in washing buffer for 2 min each time, then incubated with Hoechst 33342 (10 mg/ml in PBS) for 15 min. Samples were examined using a confocal laser-scanning microscope (Zeiss LSM 710 META, Jena, Germany) with a 40x water-immersion objective lens for fixed oocytes. Quantification of actin mesh density was carried out using a single section in the center of the oocyte image acquired by a 63x oil-immersion objective lens. Cytoplasmic regions devoid of cortical actin and actin-rich spindle were selected as square (10 μm × 10 μm), then average greyscale of phalloidin channel in the selected region were measured using imageJ. To normalize variations in phalloidin intensity between experimental repeats (n = 3), intensity values of each oocyte were normalized as percent of mean values of control group of each experimental repeat.

### Western blot analysis

A total of 100 mouse oocytes were placed in SDS sample buffer and heated at 100 °C for 5 min. Proteins were separated by SDS-PAGE and transferred to polyvinylidene fluoride membranes and probed with a goat anti-Tmod3 antibody and anti-actin antibody. As secondary antibody, HRP-conjugated goat anti-goat-IgG (1:1000; Santa Cruz Biotech, Santa Cruz Biotechnology, CA) was used. Signals were detected using Pierce ECL western blotting substrate (Thermo Fisher Scientific, Rockford, IL).

### Time-lapse microscopy of oocyte maturation

Time-lapse imaging was performed on oocytes in which Tmod3 was knocked down or overexpressed. dsRNA or cRNA was microinjected into GV-stage oocytes, as described above. For visualization of chromosomes, H2B–mCherry mRNA was also injected. Images were captured at 300 s intervals, for 7–8 h, using Lumascope 620 (Etaluma Inc, Carlsbard, CA) inverted microscope installed inside an incubator maintained at 37 °C and 5% CO_2_.

### Chromatin tracking

To track the spindle, single multicolor z slices were aligned based on the differential interference contrast channel for removal of sample drift, using a modified version of the StackRegJ plugin^66^ for ImageJ^67^. The position of chromatin was tracked using the MTrackJ plugin^68^ for ImageJ, using the RFP channels of images processed by segmented thresholding. The distances between the positions of chromatin at the starting point and at subsequent time points were recorded.

### Fluorescence recovery after photobleaching experiments

Fluorescence recovery after photobleaching (FRAP) was done by a confocal laser-scanning microscope (Zeiss LSM 710 META, Jena, Germany) with a 4X zoom of 63x/1.40 oil-immersion objective lens. For FRAP measurements, UtrCH-mCherry mRNA was injected at GV stage and incubated for 9 hr, and FRAP measurement was performed at MI stage. FRAP was carried out with 2 pre-bleach scans followed by a laser power of 100% of the 543 nm laser line, point-bleach function with full power for 5.508 s and 48 post-bleach scans. Images were taken in 512*512 format and frames were acquired at 0.97 sec/frame speed. The fluorescence intensity was measured directly acquisitions in the photo-bleached region. In order to minimize error, each oocyte measured different 3 points and then averaged. Intensity of measurement was normalized based on the pre-bleach point intensity.

### Data analysis

At least three replicate experiments were performed for each treatment. Statistical analyses were conducted using Welch’s t-test, Pearson’s chi-square test, Fisher’s exact test, or an analysis of variance (ANOVA), followed by Tukey’s multiple comparisons of means by R (R Development Core Team, Vienna, Austria). Data are expressed as the mean ± S.E.M., and P < 0.05 was considered significant.

## Additional Information

**How to cite this article**: Jo, Y.-J. *et al.* Tropomodulin-3 is essential in asymmetric division during mouse oocyte maturation. *Sci. Rep.* **6**, 29204; doi: 10.1038/srep29204 (2016).

## Supplementary Material

Supplementary Movie 1

Supplementary Movie 2

Supplementary Movie 3

Supplementary Movie 4

Supplementary Movie 5

Supplementary Movie 6

Supplementary Information

## Figures and Tables

**Table 1:** Primers used in this study.

Gene	Accession no.	Primer sequence	Use of the primer
Tmod3	NM_016963.2	5’- CAAGCATTGGAGCACAAAGA-3’	qPCR (Forward)
		5’- ACATTGGGAAAACGCTCTTG -3’	qPCR (Reverse)
		5’- TAATACGACTCACTATAGGGTTCTGTGATGTGCTGGGAAG-3’	dsRNA (Forward)
		5’- TAATACGACTCACTATAGGGCTCCTTCAATTCGCCTCTTG-3’	dsRNA (Reverse)

dsRNA; double-stranded RNA; qPCR, quantitative PCR.
