# Supplementary material for: Tropomodulin-3 is essential in asymmetric division during mouse oocyte maturation
Source: Sci Rep. 2016 Jul 4;6:29204. doi: 10.1038/srep29204 (PMC4931587; doi:10.1038/srep29204)
Supplement: Supplementary Information [file srep29204-s7.pdf]

Supplementary Informations for

Tropomodulin-3 is essential in asymmetric division during mouse oocyte maturation

Yu-Jin Jo, Woo-In Jang, Nam-Hyung Kim* and Suk Namgoong*

Department of Animal Sciences, Chungbuk National University, Cheong-Ju, ChungChungBuk-do, Republic of Korea

*Correspondence to suknamgoong@chungbuk.ac.kr or nhkim@chungbuk.ac.kr

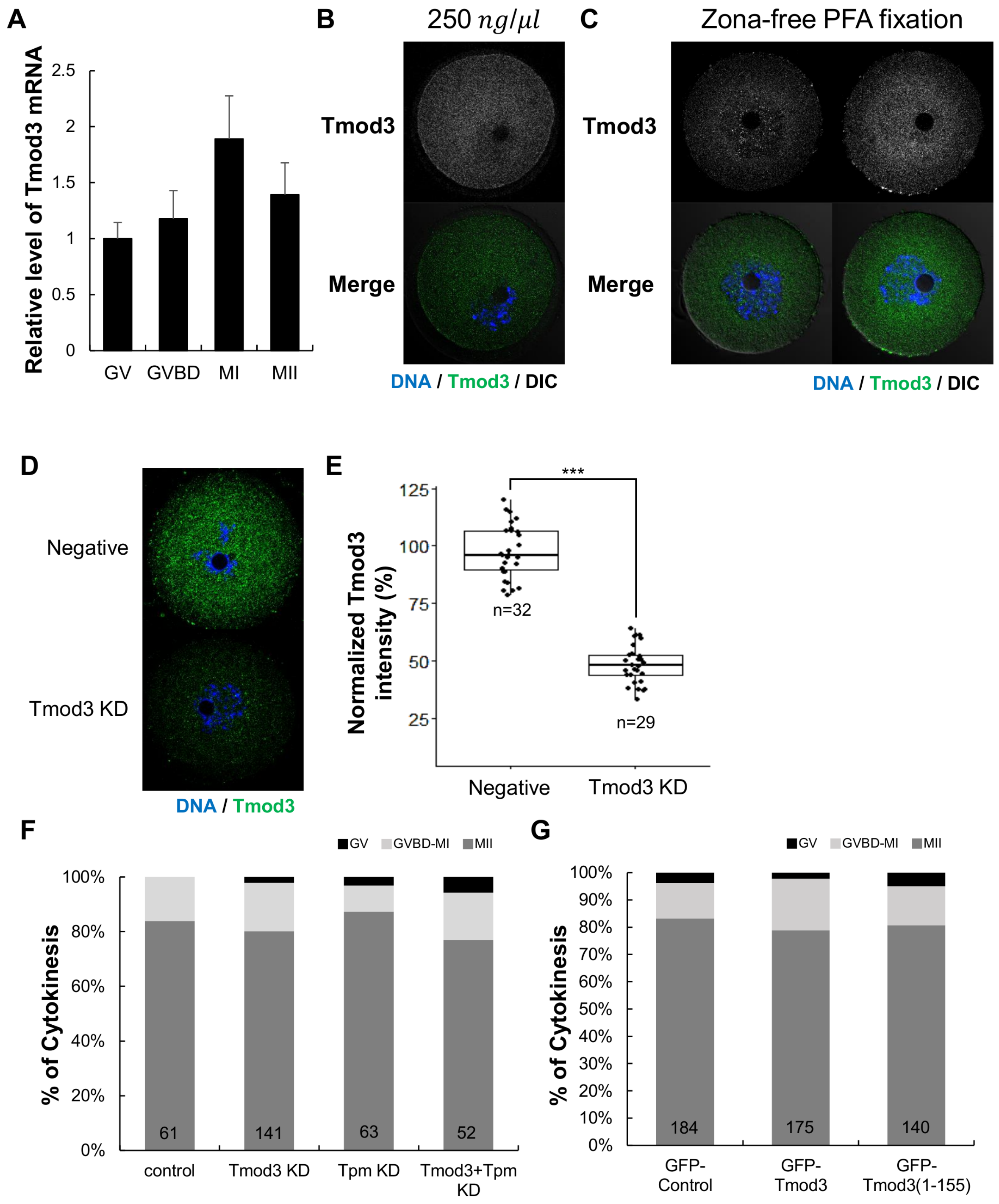

Supplementary Fig. 1. A. Relative mRNA expression levels of Tmod3 during mouse oocyte meiotic maturation. mRNA levels were measured by quantitative real-time reverse transcription PCR (qRT-PCR). Tmod3 expression levels at each stage of oocyte maturation are shown as relative fold changes compared with levels in GV-stage oocytes. Samples were collected at 0, 4, 8, or 12 h after resumption of maturation, when most oocytes were at the GV, GV breakdown (GVBD), and

metaphase MI and MII stages, respectively. Data show the mean±S.E.M. For three independent experiments. **B.** GFP-Tmod localization injected with oocyte. Same with Fig 1D, but injected with half-concentration (5-10pl of 250ng/ul of GFP-Tmod3 cRNAs) were injected. Note that weak localization of GFP-Tmod3 on cortical regions, as similar with Fig 1D. **C.** Immunostaining of Tmod3 with zona pellucida removed oocyte. Zona pellucida was removed by the treatment of tyrode's solution, then fixation, permeabilization and other immuostaining was carried out same with those in Fig 1B. **D and E**. Immunostaining of Tmod3 confirmed Tmod3 protein level depletion by dsRNA injection. Relative intensity level of Tmod3 significantly decreased in Tmod3 KD oocyte(n=29) compared with control (Negative, n=32) at the GV stage. Fluorescence intensity of each oocytes were quantified. Boxes show the interquartile range; whiskers show 1.5× the interquartile range; line represents the median. ***: P<0.001. **F and G**. Maturation status of oocytes injected with dsRNA (D: Tmod3, Tpm3.1 or Tmod3+TPm3.1) or cRNA (E: GFP, GFP-Tmod3 or GFP-Tmod3$_{1\text{-}155}$). Maturation ratio after 12hr of meiosis resumptions were examined. Total number of oocytes used for the experiments were indicated on the bar. GV: Black; GVBD/MI:Light Grey; MII:Dark Grey. Note that oocytes yield large polar body or asymmetric division is also counted as MII.

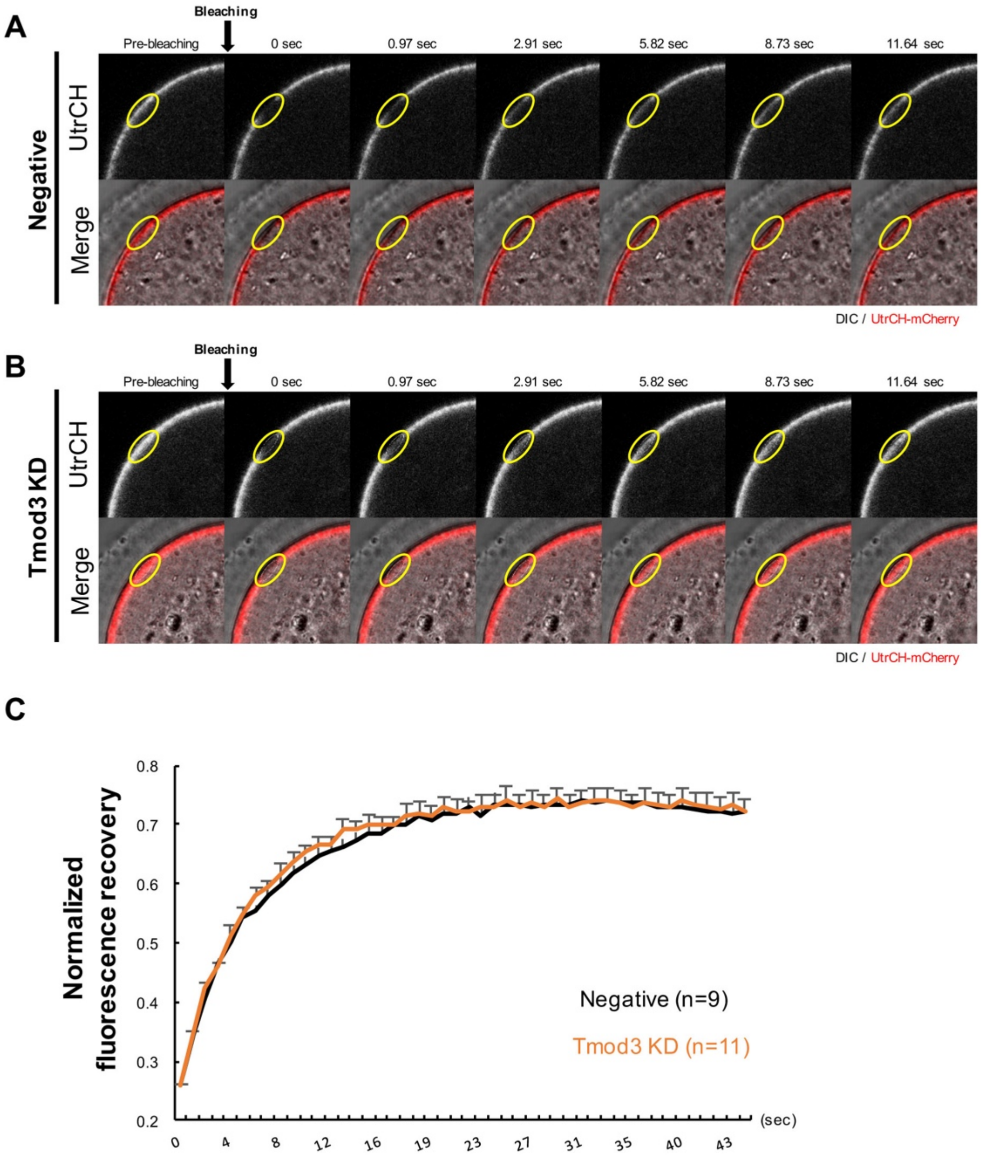

Supplementary Fig. 2. Fluorescence Recovery After Photobleacing (FRAP) in the cortex of MI oocytes. (A and B) Changes in bleached cortical actin regions in control and Tmod3 Knockdown oocytes. Actin was visualized by injection of cRNA encoding mCherry fused UtrCH. See also supplementary movies 1 and 2. (C) Time course of normalized fluorescence recovery intensity after photobleaching. In order to minimized error, each oocyte measured different three part of cortical regions. The number of measured oocyte are as indicated.

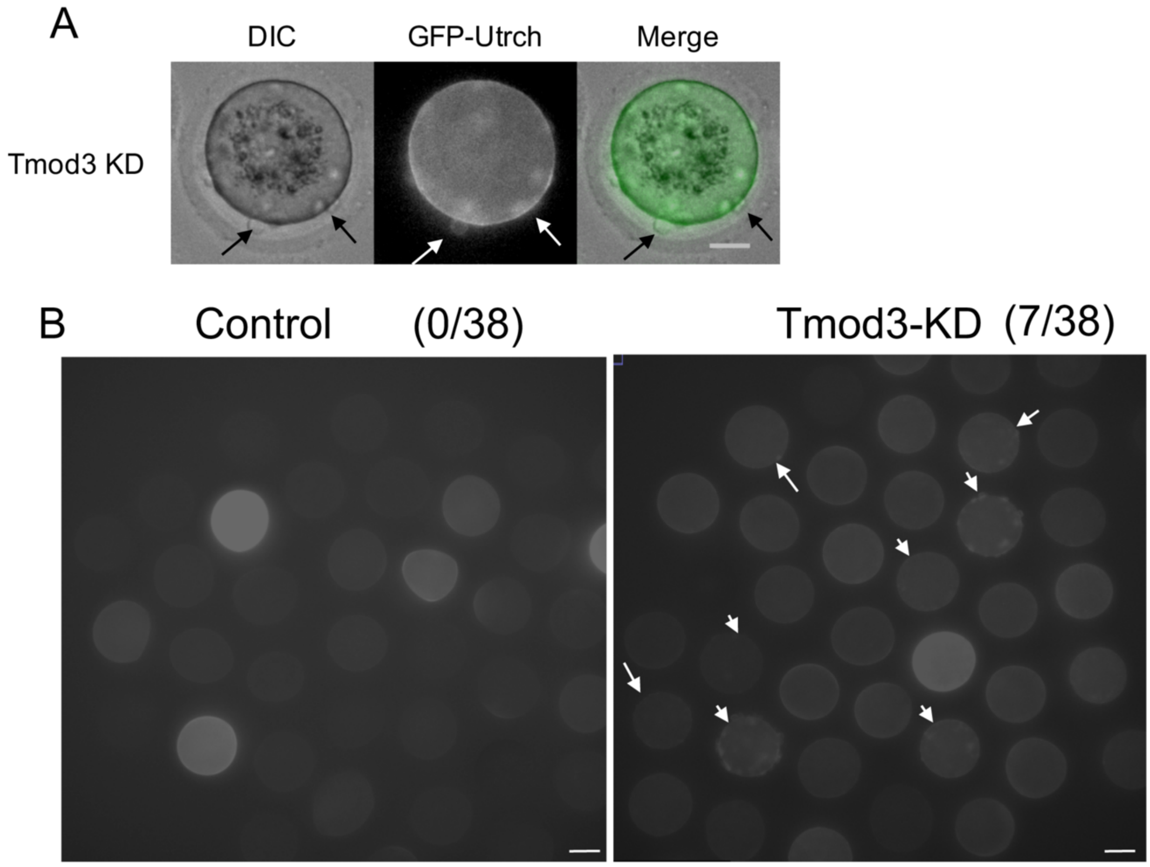

Supplementary Fig. 3. (A) Formations of membrane bleb in Tmod3 Knockdowned oocyte. Some of oocytes injected with GFP-UtrCH as well as Tmod3 dsRNA formed membrane bleb (Marked as arrow). Scale bar: 20μm. (B) Comparison of control oocytes injected with control oocytes and Tmod3 knockdown oocyte. Both oocytes were injected with GFP-UtrCH. While none of control oocyte formed membrane bleb during maturation(0/38), membrane blebs were appeared on 18% (7/38) of Tmod3 dsRNA injected oocytes. Arrow indicates blebs appeared oocytes.

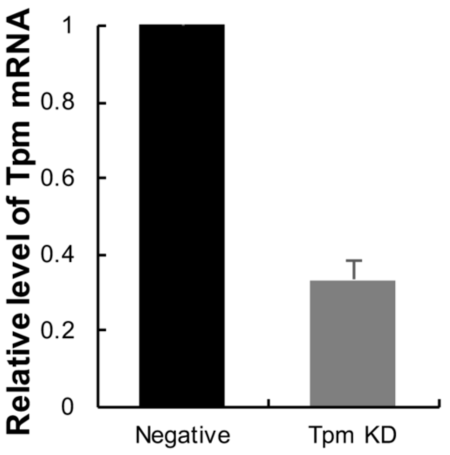

Supplementary Fig. 4. Knockdown of Tpm3,1 mRNA by siRNA injection. mRNA levels in siRNA-injected oocytes (n=30) are expressed relative to those in negative control siRNA-injected oocytes. Data indicate the mean±S.E.M. for three independent experiments.

Supplementary movie 1 and 2. Time-lapse movie of Fluorescence Recovery After Photobleacing (FRAP) in the cortical actin region. Experimented oocyte injected with UtrCH-mCherry complementary RNA (cRNA) (Negative) or Tropomodulin 3 double-stranded RNA (dsRNA) and UtrCH-mCherry cRNA (Tmod3 KD). Images were taken during 45.591 sec at MI stage oocyte. After 2 pre-bleach scans, full-power laser photobleaching was performed during 5.508sec and then fluorescence recovery was measured. After bleaching, the frame interval is 0.97sec and the total number of frame is 49 scans. Left: differential interference contrast (DIC), middle: UtrCH-mCherry (gray), which labels actin, and right: merged.

Supplementary movie 3 and 4. Time-lapse movie of an oocyte injected with UtrCH-GFP complementary RNA (cRNA) (Negative) or Tropomodulin 3 double-stranded RNA (dsRNA) and UtrCH-GFP cRNA (Tmod3 KD). Images were taken 2- 11h after NEBD. Cytoplasmic actin level increased gradiently in control oocyte (UtrCH-GFP injected). However, actin levels of cytoplasm slowly increase or impaired in Tmod3 KD oocyte compared to control groups. The frame interval is 10 min and the total length of the movie is 9h (540 min). Left: differential interference contrast (DIC), Middle: actin labeled with UtrCH-GFP (gray), which labels actin, and right: merged. Scale bar: 20 μ

m.

Supplementary movie. 5 and 6. Time-lapse movie of an oocyte injected with H2B-mcherry complementary RNA (cRNA) (Negative) or Tropomodulin 3 double stranded RNA (dsRNA) and H2B-mcherry cRNA (Tmod3 KD). Images were taken 0-12 h after the resumption of maturation, which was initiated by the removal of milrinone from the medium. Chromosome migration is almost completed at 10 h in control oocyte (H2B-mcherry injected). However, chromosome migration impaired in Tmod3 KD oocyte. The frame interval is 15 min and the total length of the movie is 720 min. Left: differential interference contrast (DIC), middle: H2B-mCherry (gray), which labels chromatin, and right: merged. Scale bar: 20 μm.

Supplementary Table 1. Primers used in this study

Gene	Accession no.	Primer sequence	Use of the primer
Tmod3	NM_016963.2	5'- CAAGCATTGGAGCACAAAGA-3'	qPCR (Forward)
		5'- ACATTGGGAAAACGCTCTTG -3'	qPCR (Reverse)
		5'- TAATACGACTCACTATAGGGTTCTGTGATGTGCTGGGAAG -3'	dsRNA (Forward)
		5'- TAATACGACTCACTATAGGGCTCCTTCAATTCGCCTCTTG -3'	dsRNA (Reverse)

dsRNA; double-stranded RNA; qPCR, quantitative PCR.
